# Antinociceptive effect of *Nephelium lappaceum* L. fruit peel and the participation of nitric oxide, opioid receptors, and ATP-sensitive potassium channels

**DOI:** 10.3389/fphar.2023.1287580

**Published:** 2023-10-31

**Authors:** Alan Santos Oliveira, Laiza Santos Biano, David Nascimento Palmeira, Damião Rabelo de Almeida, Mônica Lopes-Ferreira, Markus Kohlhoff, Jordano Augusto Carvalho Sousa, Geraldo Célio Brandão, Ana Mara de Oliveira e Silva, Renata Grespan, Enilton Aparecido Camargo

**Affiliations:** ^1^ Health Sciences Graduate Program, Federal University of Sergipe, Aracaju, Brazil; ^2^ Physiological Sciences Graduate Program, Federal University of Sergipe, São Cristóvão, Brazil; ^3^ Department of Physiology, Federal University of Sergipe, São Cristóvão, Brazil; ^4^ Immunoregulation Unit of the Laboratory of Applied Toxinology (CeTICs/FAPESP), São Paulo, Brazil; ^5^ Oswaldo Cruz Foundation, René Rachou Institute, Belo Horizonte, Brazil; ^6^ Department of Pharmacy, Federal University of Ouro Preto, Ouro Preto, Brazil; ^7^ Department of Nutrition, Federal University of Sergipe, São Cristóvão, Brazil

**Keywords:** *Nephelium lappaceum*, fruit peel, antinociception, locomotor activity, toxicity

## Abstract

**Introduction:** *Nephelium lappaceum* L. (Sapindaceae) is a plant known as rambutan. It is used for various purposes in traditional medicine.

**Objective:** We aimed to evaluate the antinociceptive effects of the ethanol extract of the fruit peel of *N. lappaceum* (EENL), the mechanisms involved in these effects, and the acute toxicity in zebrafish.

**Methods:** We performed chromatography coupled to mass spectrometry, acute toxicity assay in zebrafish, and evaluation in mice submitted to models of nociception and locomotor activity.

**Results:** We identified (epi)-catechin, procyanidin B, and ellagic acid and its derivatives in EENL. We did not find any toxicity in zebrafish embryos incubated with EENL. The locomotor activity of mice submitted to oral pretreatment with EENL was not changed, but it reduced the abdominal constrictions induced by acetic acid, the licking/biting time in both the first and second phase of formalin testing and capsaicin testing, and carrageenan-induced paw mechanical allodynia. Oral pretreatment with EENL increased latency time in the hot plate test. This antinociceptive effect was significantly reversed by naloxone, L-arginine, and glibenclamide respectively showing the participation of opioid receptors, nitric oxide, and KATP channels as mediators of EENL-induced antinociception.

**Conclusion:** EENL causes antinociception with the participation of opioid receptors, nitric oxide, and KATP channels, and is not toxic to zebrafish.

## 1 Introduction

The International Association for the Study of Pain defines pain as “an unpleasant sensory and emotional experience associated with, or resembling that associated with, actual or potential tissue damage” (Raja et al., 2020). It can result from the direct activation and sensitization of the primary sensory neurons by autacoids such as prostaglandin, proinflammatory cytokines, and chemokines. In this case, it is known as inflammatory pain (Baral et al., 2019). It can also arise from central sensitization, independent of the stimulation of the sensory terminals (Nijs et al., 2015).

Treatment of pain is still a challenge since the use of non-steroidal anti-inflammatory drugs causes deleterious gastrointestinal, hepatic, renal, and cardiovascular effects, and opioids, although widely used, can induce constipation, pharmacological tolerance, and addiction, among other severe side effects (Carter et al., 2014).

Considering these aspects, plants possessing antinociceptive activity have been the target of many researchers seeking bioactive compounds as new alternatives with better therapeutic responses, and if possible, without intense side effects. In this sense, *Nephelium lappaceum* L., from the Sapindaceae family, is a plant that arouses interest. It is popularly known as rambutan or hairy litchi, and is native to Malaysia, but can now be found in Brazil and other parts of the world (Junior et al., 2016; Sukmandari et al., 2017; Mahmood et al., 2018; Afzaal et al., 2023).

In traditional medicine, *N. lappaceum* is used to treat diarrhea, stomachache, dysentery, giardiasis, fever, and skin diseases (Sukmandari et al., 2017; Mahmood et al., 2018; Afzaal et al., 2023). Its fruit is used to treat fever, severe dysentery, and diarrhea, for its carminative effect in dyspepsia as well as for its astringent, anthelmintic, and antidiabetic activities. Root decoctions are often consumed to relieve fever, while leaves are used as poultices for headaches and bark preparations as astringents (Sukmandari et al., 2017; Mahmood et al., 2018). In Malaysia, dried rambutan fruit rind is traditionally employed, mainly for diabetes and high blood pressure (Palanisamy et al., 2008; Sukmandari et al., 2017; Albuquerque et al., 2023).

Several studies have investigated the biological effects of *N. lappaceum*. The extract of rambutan peel has been found to have potential antidiabetic and antihypercholesterolemia effects (Palanisamy et al., 2011) Methanol seed extract is reported to have significant antidiarrheal activity (Morshed et al., 2014).

Regarding nociception and/or associated inflammation, Kumar et al. (Kumar et al., 2012) reported the anti-arthritis effect of ethanol extract of the rind of *N. lappaceum* in the model of collagen-induced arthritis in rats but did not evaluate its effect on nociception. Treatment with the ethanol extract of *N. lappaceum* leaves decreased paw biting/licking number in the first and second phases of formalin testing (Subramaniam et al., 2015). Morshed et al. (Morshed et al., 2014) demonstrated that the administration of methanol extract from seeds of *N. lappaceum* at doses between 500 and 2000 mg/kg did not induce mortality in mice. In addition, they reported antinociceptive and anti-inflammatory activity, indicated by a decreased number of abdominal writhes induced by acetic acid, the number of paw biting/licking in the first and second phases of the paw formalin test, and reduced carrageenan-induced paw edema.

No studies have focused on the antinociceptive activities of *N. lappaceum* fruit peel, but it is of interest based on the potential of the constituents commonly found in this part of the plant, like ellagic acid, corilagin, geraniin and other phenolic compounds (Peixoto Araujo et al., 2021). Therefore, we evaluated the antinociceptive activity and mechanisms underlying the action of the ethanol extract of *N. lappaceum* in pre-clinical models in rodents, as well as evaluated whether this extract induces any acute toxicity in zebrafish.

## 2 Methods

### 2.1 Obtaining and characterizing the ethanol extract of the fruit peel of *N. lappaceum* (EENL)

A specimen from *N. lappaceum* was collected in January 2023 in the municipality of Valença, Bahia state, Brazil (13°29′46.0″S 39°12′32.5″W) and deposited in the Herbarium of the Federal University of Sergipe (ASE42843), where it was identified by Dr. Marla I. U. Oliveira.

Fresh *N. lappaceum* fruits were pulped at room temperature and only ripe fruits with no visible signs of damage were utilized. For the preparation of the ethanol extract, approximately 50 g of the lyophilized fruit peel was weighed and diluted in 200 mL of absolute ethanol followed by magnetic stirring for 24 h. Subsequently, the mixture was centrifuged for 15 min, and the supernatant was vacuum-filtered and dried to obtain EENL (10.5 g). The extract was kept in amber glass bottles tightly closed under refrigeration until use in the experiments.

### 2.2 Reversed-phase ultra-high-performance liquid chromatography (RP-UHPLC) coupled to diode array detection (DAD) and mass spectrometry (MS) analysis of ethanol extracts of the fruit peel of *Nephelium lappaceum* L.

LC-MS/MS analyses were carried out with a Nexera UHPLC system (Shimadzu) hyphenated to a maXis ETD high-resolution ESI-QTOF mass spectrometer (Bruker), controlled by the Compass 1.7 software package (Bruker). Samples of EENL were injected in a Shimadzu Shim-Pack XR-ODS-III column (C18, 2.2 um, 2.0 × 150 mm) at 40°C and 400 μL/min. The mobile phases were A (0.1% formic acid in ultrapure water) and B (acetonitrile) and they formed an initial eluent gradient of 0.5 min 5% B, a linear gradient to 100% B in 10 min and a hold at 100% B for 1.5 min.

The acquisition of the mass spectra, the mass calibration, and compound detection and identification were performed as previously described (Ceravolo et al., 2018).

### 2.3 Acute toxicity test with zebrafish

Adult zebrafish with age (*<*18 months old) of the AB strain (International Zebrafish Resource Center, Eugene, OR) were maintained apart by sex and reared under standard conditions (28°C, pH 7, and light/dark cycle of 14/10 h) in individual aquariums (Alesco, Campinas, Brazil) using synthetic brine (60 μg/mL of Instant Ocean sea salts). The experiments were approved by the Butantan Institute Animal Use Ethics Commission (CEUAIB #6438210220) and were conducted in fertilized embryos exposed to E2 medium without or with different EENL concentrations (0.95 and 1.9 μg/mL, or 100 and 200 mg/kg equivalent, respectively), following the protocol of OECD 236: Fish Embryo Acute Toxicity Test and the previous description (Henrique et al., 2021).

### 2.4 Tests of nociception and locomotor activity in mice

Tests of nociception and locomotor activity were conducted in male Swiss mice weighing 20–30 g. They were obtained from the Animal Center of the Federal University of Sergipe and were maintained at 21°C–23°C with free access to food and water under a 12-h light/dark cycle. The experiments were approved by the Ethics Committee on Animal Use of the Federal University of Sergipe on 4 December 2019 (# 1122270819). Quantification of nociceptive behavior and locomotor activity was carried out by a trained investigator who was blinded to the group’s identity.

### 2.5 Evaluation of locomotor activity (open field test)

Animals were treated with EELN (200 mg/kg, 1 h before, p.o.), vehicle (0.9% saline, p.o.), or diazepam (1.5 mg/kg; 30 min before, s.c.) and placed individually in an open field (*n* = 8). The analysis was performed according to the previous description (Capaz et al., 1981) with a camera positioned over the open field at a height of 200 cm and connected to a computer equipped with an animal tracking program (Anymaze^®^, Stoelting, United States) to record behavioral parameters.

### 2.6 Noxious stimuli-induced nociception

For all tests of the nociception procedure, animals were pretreated with EENL (50, 100, or 200 mg/kg, p.o.) based on Kumar et al. (2012) or vehicle (saline 0.9%, p.o.) 1 h before stimulation.

The abdominal constrictions induced by acetic acid were performed as previously described (Collier et al., 1968). Abdominal writhes were induced in mice by injection of acetic acid (0.6%, 0.1 mL/10 g, i.p.). A control group received acetylsalicylic acid (ASA, 300 mg/kg, 1 h before, p.o., *n* = 6/group). The abdominal writhes were counted for 20 min, beginning from 5 min after injection of the acetic acid.

The formalin test was conducted according to Hunskaar and Hole (Hunskaar and Hole, 1987). Control groups received morphine (10 mg/kg, 30 min before, s.c.) or ASA (300 mg/kg, 1 h before, p.o.). Formalin solution (1%, 20 μL, intraplantar) was injected in the right hind paw of each animal (*n* = 6–7/group). The paw licking or biting time was measured for each mouse from 0 to 5 min (first phase) and from 15 to 30 min (second phase) after formalin injection.

The acute inflammatory carrageenan-induced mechanical allodynia was performed as previously described (Cunha et al., 1992). Animals were transferred randomly to individual cages (dimensions 20 × 22 × 18 cm) once a day for 30 min for 3 days for acclimatation. Before any treatment, the basal nociceptive behavior was obtained as the average of three measures of the intensity of the mechanical stimulus necessary for the removal of the paw (baseline measure), by using an electronic von Frey device. After this measurement, animals (n = 8) were treated with EENL, vehicle, or indomethacin (15 mg/kg; control group, i.p.). After 1 h, 20 μL of carrageenan (300 μg/paw) was administered and the intensity of the mechanical stimulus was evaluated 4 h later. The values were expressed as the stimulus intensity (g).

Capsaicin-induced nociception was performed according to a previous study (Sakurada et al., 1992) with *n* = 8 mice. Morphine (10 mg/kg, 30 min before induction, i.p.) was used as control. Animals were submitted to a subplantar injection of capsaicin (20 μL, 1.6 µg/paw), and were observed for each mouse for 5 min after the injection of capsaicin to evaluate their time of biting or licking the injected paw.

#### 2.6.1 Thermal nociception test (hot plate)

Hot plate test was performed as previously described (Jacob and Ramabadran, 1978). Animal groups (*n* = 8) were placed individually on a metal plate heated to 55.0ºC ± 0.5°C. We measured the latency time (in seconds) to the appearance of reactions to the thermal stimulus, such as licking, tapping, or flicking the back leg. They were subsequently treated with vehicle (0.9% saline, p.o.), EELN (50, 100, or 200 mg/kg, p.o.) or morphine (10 mg/kg, i.p.) 30 min before, and at 30, 60, and 120 min after treatment the measurement in the hot plate was repeated. Animals that stayed up to 30 s on the plate were retrieved to prevent damage. The area under the curve (AUC _[0–2 h]_) was calculated using the trapezoidal rule and was used as an integrative index of nociceptive activity. Twenty-4 hours before the test, animals that remained on the plate for more than 10 s without reaction to the stimulus were excluded.

Possible mechanisms underlying EENL action were investigated in additional sets of experiments. To investigate the role of nitric oxide (NO) in the antinociception caused by EENL, mice (*n* = 8/group) were pretreated with L-arginine (600 mg/kg, i.p., a NO precursor), and after 15 min they received EENL (100 mg/kg, p.o.), N(ω)-nitro-L-arginine methyl ester (L-NAME; 100 mg/kg, i.p., a NO synthase inhibitor), or vehicle (saline 0.9%, p.o.) (Beirith et al., 1998). Then the animals were subjected to the hot plate test as previously described.

The contribution of ATP-sensitive potassium (K_ATP_) channels or opioid receptors was investigated respectively by the pretreatment with mice with glibenclamide (a K_ATP_ channel inhibitor, 10 mg/kg, i.p.) or naloxone (2 mg/kg, s.c.). After 15 min, mice received EENL (100 mg/kg, p.o.) or vehicle (saline 0.9%, p.o.) (Perimal et al., 2011) and were subjected to the hot plate test as described above.

### 2.7 Statistical analysis

Data were shown as means ± SEM. The Shapiro-Wilk test was used to ensure the normality of the data. One- or two-way analysis of variance (ANOVA) followed by Tukey´s test was used to detect the differences among the groups, which were considered significant when *p*-values were lower than 0.05.

## 3 Results

### 3.1 Characterization of EENL by RP-UHPLC-DAD analysis

**FIGURE 1:**
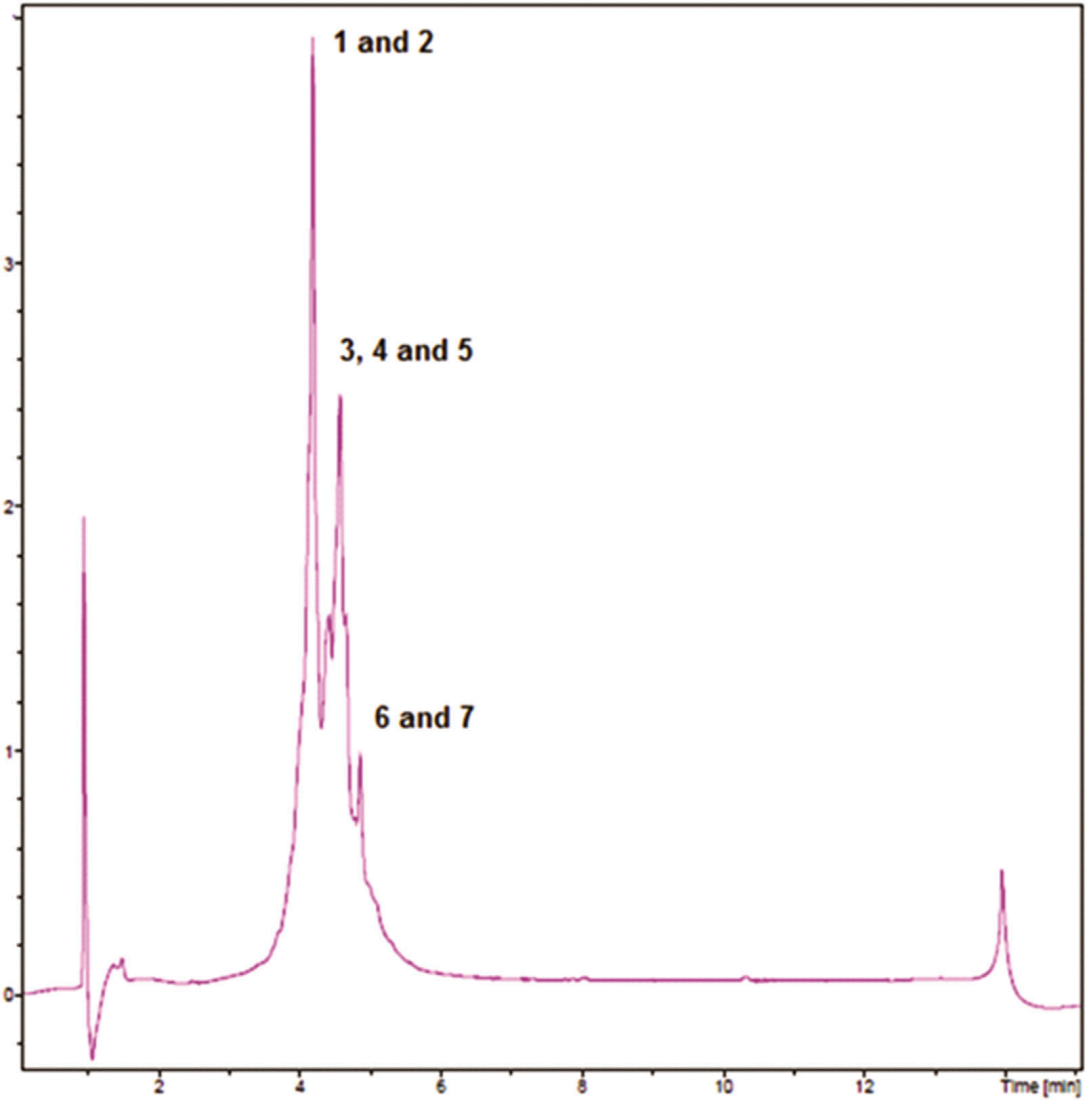
RP-UHPLC-DAD profiles of EENL.

Figure 1 shows the RP-UHPLC-DAD profile of EELN. We identified the seven major peaks of this chromatogram as procyanidin B (Raja et al., 2020), (epi)-catechin (Baral et al., 2019), ellagic acid derivative (punicalin) (Nijs et al., 2015), ellagic acid derivative (Carter et al., 2014), ellagic acid derivative (pedunculagin) (Junior et al., 2016), ellagic acid-4-O-xylopyranoside (Sukmandari et al., 2017) and ellagic acid (Mahmood et al., 2018) (Table 1).

**TABLE 1:** Phenolics identified in ethanol extract of the fruit peel of *N. lappaceum* (EENL) by LC-DAD-HRMS.

Peak	Compound	Molecular formula	UV (nm)	LC-MS [M + H]^+^ (*m/z*) (MS MS)	LC-HRMS [M + H]^+^ (*m/z*)
1	Procyanidin B	C_30_H_26_O_12_	220; 278	579.1497 (291.0865; 289.0704; 287.0548; 271.0596; 247.0601; 163.0396; 139.0394; 135.0443; 127.0390)	579.1491
2	(Epi)-catechin	C_15_H_14_O_6_	220; 279	291.0859 (165.0547; 139.0393; 123.0444)	291.0860
3	Ellagic acid derivative (Punicalin)	C_34_H_22_O_22_	220; 277	783.0671 (463.0504; 337.0186; **303.0132**; 277.0339; 275.0184; 235.0235)	783.0671
4	Ellagic acid derivative	C_40_H_28_O_27_	220; 279	941.0848 (477.0288; **303.0139**; 277.0341; 275.0164; 221.0077; 193.0132)	941.0887
5	Ellagic acid derivative (Pedunculagin)	C_34_H_24_O_22_	220; 278	785.0791 (339.0346; **303.0138**; 277.0342; 275.0189; 221.0078)	785.0817
6	Ellagic acid-4-O-xylopyranoside	C_19_H_14_O_12_	220; 278	435.0549 (**303.0130**; 285.0019; 275.0189; 257.0074; 247.0225; 229.0119; 201.0178)	435.0551
7	Ellagic acid	C_14_H_6_O_8_	220; 279	303.0132 (285.0044; 275.0183; 257.0077; 247.0233; 229.0126; 201.0186; 173.0231; 145.0282)	303.0130

### 3.2 Incubation with EENL did not cause toxicity to zebrafish

**FIGURE 2:**
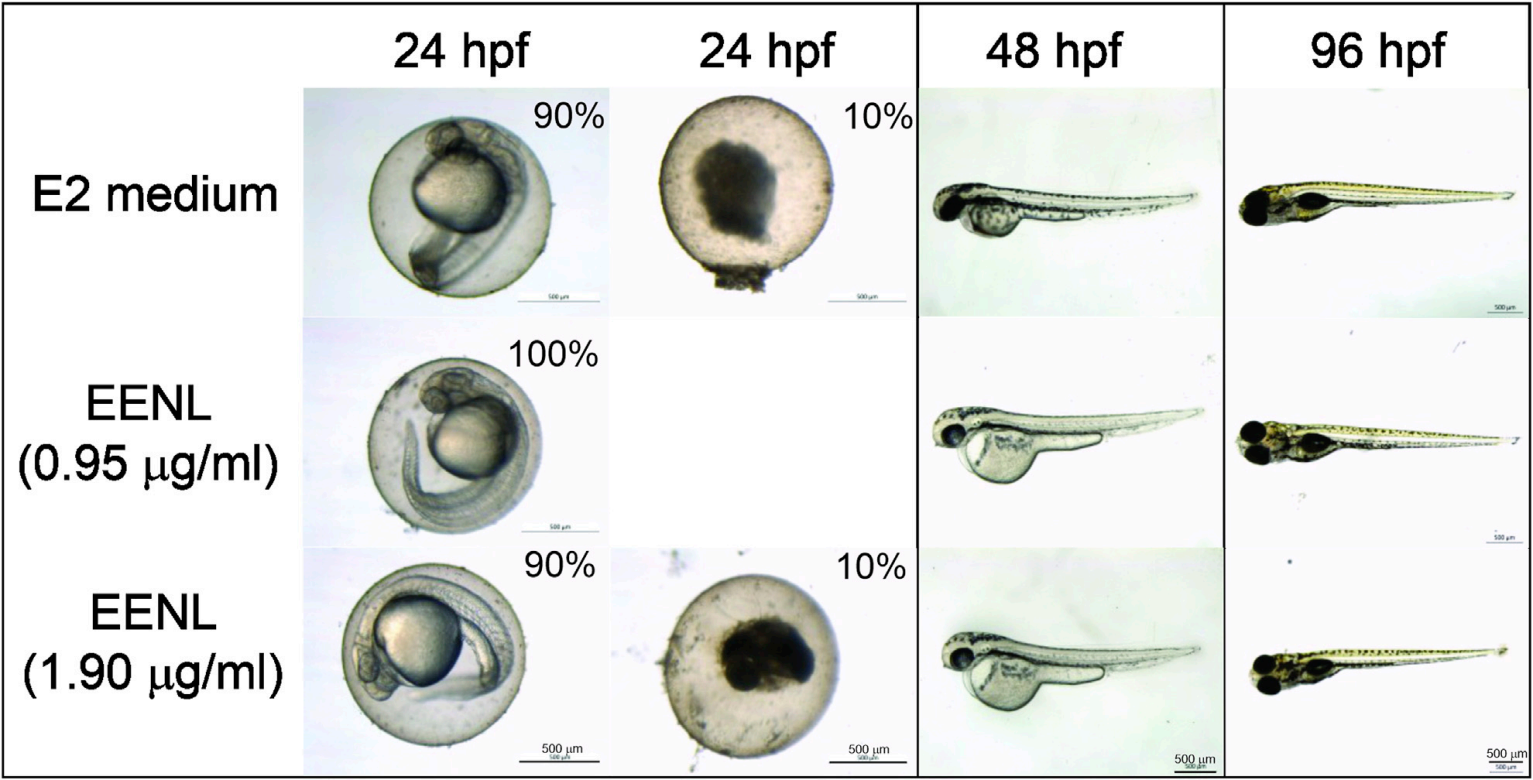
Representative images of zebrafish exposed to EENL at different concentrations up to 96 h post-fertilization (hpf). Mortality of 10% was observed for both E2 medium and EELN at 1.9 μg/mL, but not at 0.95 μg/mL after 24 h.

Acute toxicity of EENL, based on zebrafish coagulation and mortality, was evaluated each 24 h until the endpoint of 96 hpf. After 24 hpf, we did not observe any mortality of zebrafish embryos incubated with EENL at 0.95 μg/mL (*n* = 20), which showed normal development up to 96 hpf. Among the embryos incubated with EENL at 1.90 μg/mL (*n* = 20), we observed 2 coagulated embryos at 24 hpf (10% mortality), but the other embryos developed normally up to 96 hpf (Figure 2). In the group of embryos incubated with E2 medium (*n* = 20), we also detected 2 coagulated embryos at 24 hpf. After incubation for 48 h, surviving embryos from all groups presented normal embryonic development, with the presence of some animals hatched from the egg. At 72 h, all embryos from the groups hatched, and at 96 hpf no morphological change and/or teratogenicity was detected in any larvae of the groups evaluated.

The EENL concentrations (0.95 and 1.9 μg/mL) did not induce changes in the survival rate in comparison with the control. In addition, EENL in the tested concentrations did not induce developmental defects at the end of the test (96 hpf).

### 3.3 Lack of locomotor effects of EENL on mice in the open field test

Table 2 shows that 1 h after administration of EENL (200 mg/kg) to animals, no significant change in the distance covered in the open field test was observed compared to the vehicle group. As a control, diazepam administration (1.5 mg/kg) reduced this distance (*p* < 0.001 vs. vehicle group).

**TABLE 2:** Effect of the ethanol extract of fruit peel of *Nephelium lappaceum* (EENL) on locomotor activity of mice.

Treatment	Distance traveled (m)
Vehicle	25.1 ± 2.1
EENL (200 mg/kg)	21.2 ± 1.6
Diazepam (1.5 mg/kg)	8.8 ± 1.7***

Data are expressed as mean ± SEM, of the distance covered in the open field, *n* = 6. One-way ANOVA, followed by the Tukey post-test. ***p < 0.001 vs. vehicle.

### 3.4 Pretreatment with EENL reduced acetic acid-induced abdominal constriction

As shown in Table 3, pretreatment of mice with EENL at 50, 100, and 200 mg/kg inhibited the abdominal constriction caused by acetic acid injection by 29.9%, 54.3%, and 63.2% respectively (*p* < 0.05, *p* < 0.001 and *p* < 0.001 compared to the vehicle group), as did acetylsalicylic acid (300 mg/kg; 64.1% inhibition; *p* < 0.001).

**TABLE 3:** Effect of the ethanol extract of the fruit peel of *Nephelium lappaceum* (EENL) on acetic acid-induced writhing.

Treatment	Number of writhes	Inhibition (%)
Vehicle	62.6 ± 6.3	—
EENL (50 mg/kg)	43.8 ± 3.9*	29.9 ± 6.3
EENL (100 mg/kg)	28.6 ± 3.6***	54.3 ± 5.7
EENL (200 mg/kg)	23.0 ± 3.5***	63.2 ± 5.6
ASA (300 mg/kg)	23.1 ± 3.4***	63.0 ± 5.4

Data are expressed as mean ± SEM, of the number of writhes, *n* = 6–8. One-way ANOVA, followed by the Tukey post-test.*p < 0.05 or ***p < 0.001 vs. vehicle.

### 3.5 Pretreatment with EENL decreased formalin-induced nociception in mice paw

**FIGURE 3:**
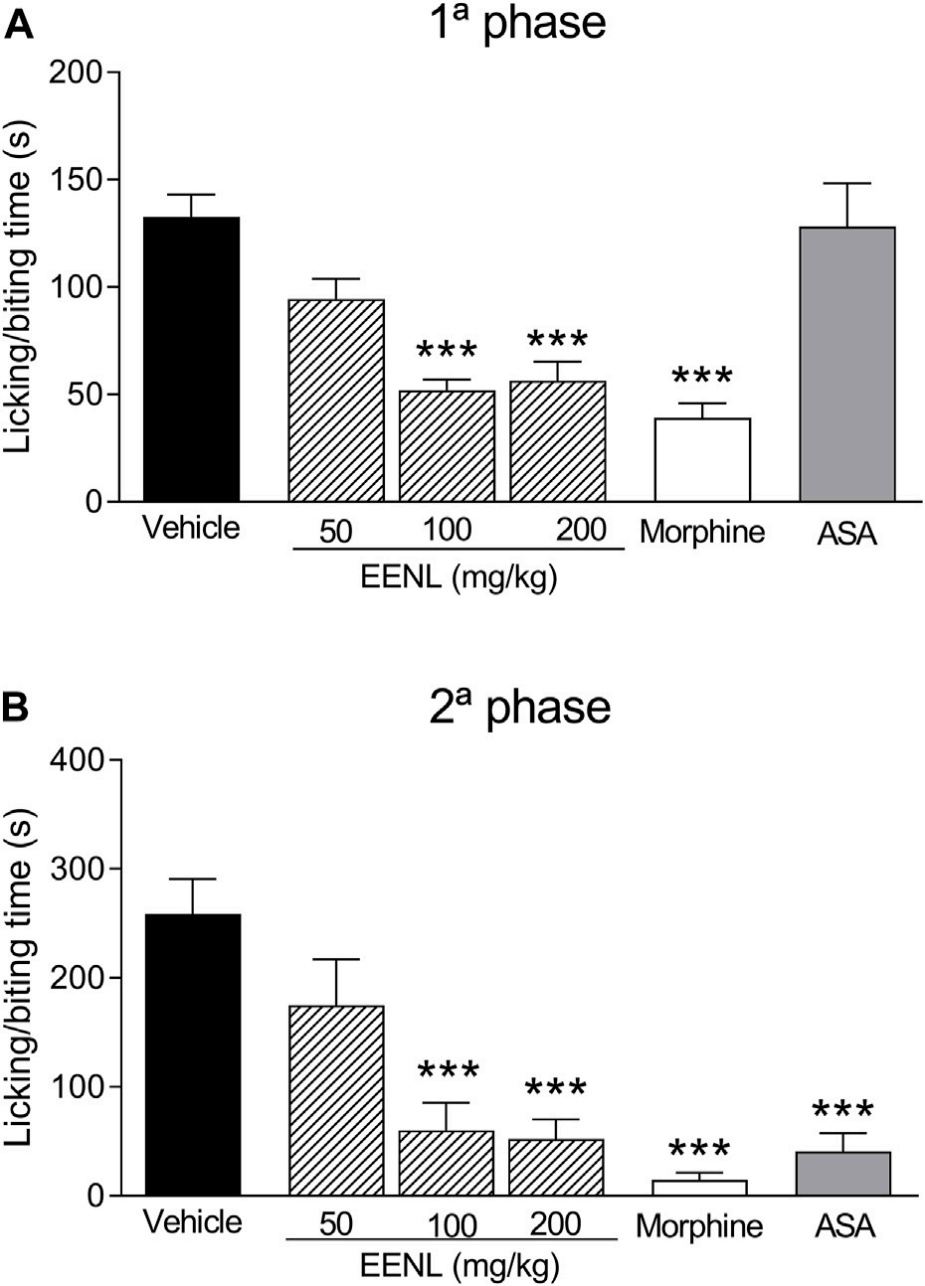
EENL reduces paw licking/biting induced by formalin in mice. Data are mean ± SEM of time (s) of the nociceptive behavior. The first phase **(A)** and the second phase **(B)** are shown (*n* = 6–7). ***p < 0.001 vs. vehicle-treated group. One-way ANOVA followed by the Tukey test.

The nociceptive behavior was observed in both the first and second phases after the injection of formalin (1%) (Figure 3). Pretreatment with EENL (100 and 200 mg/kg) diminished the licking/biting time in both the first (*p* < 0.001 each, Figure 3A) and second phases (*p* < 0.001 each, Figure 3B), in comparison with animals pretreated with vehicle. Morphine significantly decreased the licking/biting time in both the first and second phases (*p* < 0.001) while acetylsalicylic acid reduced this parameter only in the second phase (*p* < 0.001).

### 3.6 Inhibitory effect of EENL on carrageenan-induced mechanical allodynia in mice paws

**FIGURE 4:**
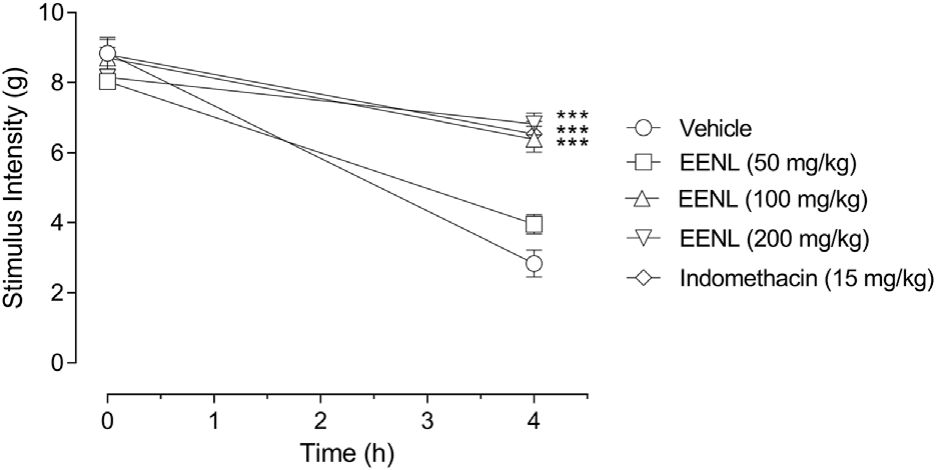
Effect of EENL on carrageenan-induced mechanical allodynia in mice paws. The stimulus intensity threshold was measured before pretreatments (basal) and 4 h after injection of carrageenan (*n* = 6–7). Two-way ANOVA followed by the Tukey test. ***p < 0.001 vs. vehicle-treated group.

Figure 4 shows that the injection of carrageenan (1%) decreased the stimulus intensity threshold applied to mice paws to elicit behavioral response in animals previously treated with vehicle (from 8.8 ± 0.5 g at basal to 2.8 ± 0.4 g at 4 h, *p* < 0.001, comparison not shown in Figure 4). In the basal measurement before any treatment, all the groups had similar values of stimulus intensity threshold. Four hours after the carrageenan injection, the groups pretreated with 100 or 200 mg/kg of EENL showed stimulus intensity threshold significantly higher than the vehicle group (*p* < 0.001 each, Figure 4). However, in the group pretreated with EENL at 50 mg/kg, no changes were observed. Animals pretreated with indomethacin (15 mg/kg) also showed augmented stimulus intensity threshold at 4 h after carrageenan injection (*p* < 0.001) compared to the vehicle group.

### 3.7 Inhibition of capsaicin-induced nociception by pretreatment with EENL

Table 4 demonstrates that pre-treatment with EENL at 100 and 200 mg/kg reduced mice’s paw licking/biting time (*p* < 0.001) after administration of capsaicin in comparison with the vehicle group, as did morphine (*p* < 0.001).

**TABLE 4:** Effect of the ethanol extract of the fruit peel of *Nephelium lappaceum* (EENL) on capsaicin-induced nociceptive response in mice paws.

Treatment	Licking/biting time (s)
Vehicle	70.3 ± 7.2
EENL (100 mg/kg)	29.4 ± 4.8***
EENL (200 mg/kg)	31.6 ± 4.1***
Morphine (10 mg/kg)	17.7 ± 2.5^***^

Data are expressed as mean ± SEM, of the licking/biting time, *n* = 7. One-way ANOVA, followed by the Tukey post-test. *p* < 0.001 vs. vehicle.

### 3.8 Treatment with EENL increased latency time of mice on hot plate

**FIGURE 5:**
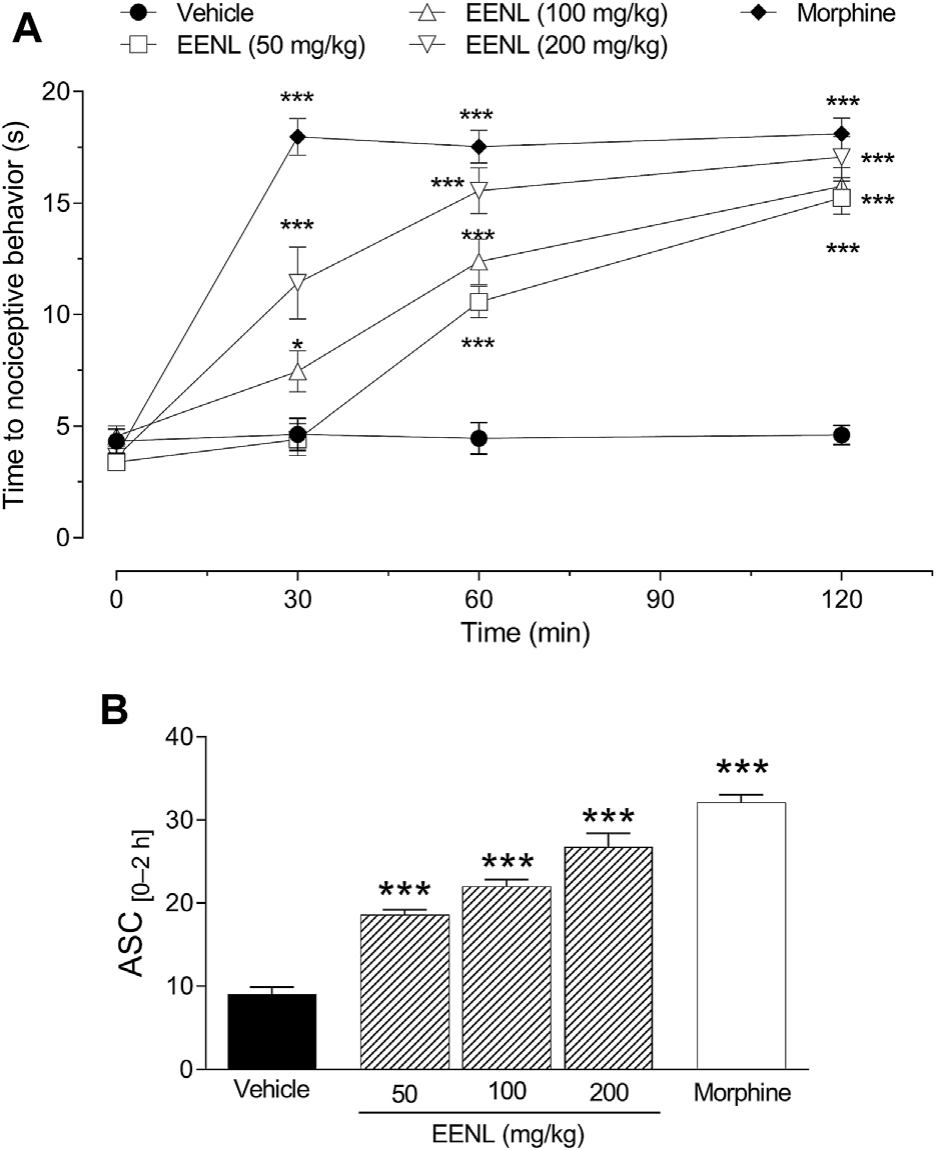
Effect of EENL on the hot plate latency time. The time for mice to display nociceptive behavior was measured before (basal) and 30, 60, and 120 min after treatment with EENL (50, 100, 200 mg/kg, p.o.) or morphine **(A)**. The areas under the curves (AUC) from 0 to 2 h are shown in **(B)**. Data are mean ± SEM, *n* = 8. *p < 0.05 or ***p < 0.001 vs. vehicle treated group. One-way **(B)** or two-way **(A)** ANOVA followed by the Tukey post-test.

After mice were placed in the hot plate, they took 4.3 ± 0.5 s to show a nociceptive response. This time did not vary significantly among different groups at baseline, nor was it altered at times of 30, 60, and 120 min after treatment (Figure 5A).

The treatment with EENL (50 mg/kg) did not change the latency time after 30 min, but at 60 and 120 min after the treatment, the latency time on the hot plate was increased (*p* < 0.001) in comparison with the vehicle group. (Figure 5A). The administration of EENL at doses of 100 and 200 mg/kg augmented the latency time of mice on the hot plate after 30 (*p* < 0.05 and *p* < 0.001 respectively), 60 (*p* < 0.001 each), and 120 min (*p* < 0.001 each) in comparison with animals that were treated with vehicle. Animals that received morphine (3 mg/kg) presented increased (*p* < 0.001) latency time at all time points evaluated (Figure 5A).

We calculated the AUC and used it as an integrating index. The groups treated with EENL (50, 100, and 200 mg/kg) or morphine (3 mg/kg) had higher AUC values (*p* < 0.001) in comparison with the group treated with vehicle (Figure 5B). Based on these results, we chose the intermediate dose (100 mg/kg) of EENL for the subsequent tests to evaluate the antinociception mechanisms.

### 3.9 Involvement of opioid receptors, NO pathway, and KATP channels in EENL-induced antinociception

**FIGURE 6:**
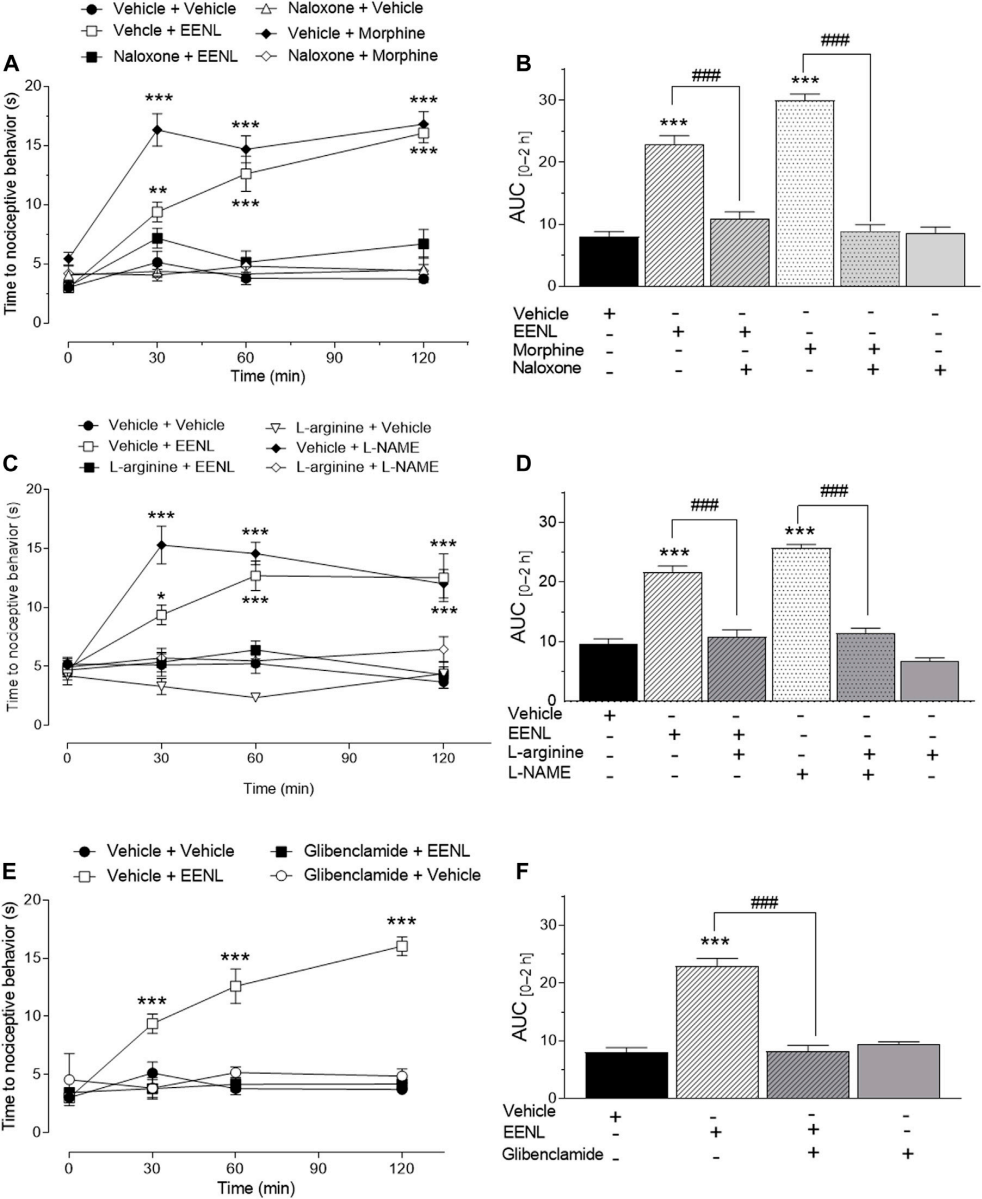
Pharmacological interpretation of the effect of EENL in the hot plate test. The effect of the opioid system **(A, B)**, L-arginine/nitric oxide pathway **(C, D)**, and ATP-sensitive K^+^ channel inhibitors (panels E and F) was verified in the antinociception caused by EENL. Each value represents the mean ± SEM of n = 5–6. **(A, C, E)** Two-way ANOVA followed by the Tukey post-test; *p < 0.05, **p < 0.01 or ***p < 0.001 vs. respective vehicle-treated group. **(B, D, F)** One-way ANOVA followed by the Tukey post-test. ***p < 0.001 vs. vehicle and ^###^ *p* < 0.001.

The participation of the opioid system in the antinociceptive activity of EENL in the hot plate test is shown in Figure 6A. The treatment with EENL (100 mg/kg) increased latency time at 30 (*p* < 0.05), 60, and 120 min (*p* < 0.001) of mice on the hot plate, as did morphine (5 mg/kg; *p* < 0.001 for each time point), in comparison with vehicle-treated mice. Pretreatment with naloxone (2 mg/kg) reversed the antinociceptive effect of EENL or morphine since no change was observed in the latency time at any time point of animals receiving naloxone before EENL or morphine in comparison with the vehicle group. The pretreatment with naloxone before vehicle administration did not change the latency time. Accordingly, the AUC (Figure 6B) was significantly higher for animals treated with EENL or morphine in comparison with their respective group pretreated with naloxone (*p* < 0.001).

Figure 6C shows the participation of NO in the antinociceptive activity of EELN in the hot plate test. It demonstrates that the increased latency time caused by treatment with EENL (100 mg/kg) or L-NAME (100 mg/kg) was reverted by pretreatment with L-arginine (600 mg/kg) since no change was detected in the latency time of the L-arginine plus EENL or L-NAME groups in comparison with the animals treated with vehicle. Administration of L-arginine followed by vehicle did not change the latency time of the animals on the hot plate at any time point evaluated in comparison with the vehicle group. The analysis of AUC (Figure 6D) confirmed that L-arginine pretreatment restored the effects of both EENL (*p* < 0.001) and L-NAME (*p* < 0.001) to the basal values, but L-arginine itself had an effect similar to that of the vehicle treated-group.

The antinociceptive effect of EENL was also reversed by the pretreatment with glibenclamide (Figure 6E), since the group pretreated with this K_ATP_ channel blocker (10 mg/kg) before EENL administration did not show alteration of the latency time after 30, 60 or 120 min on the hot plate, in comparison with the vehicle group, while EENL increased this time (*p* < 0.001). This result was corroborated by the calculation of the AUC of glibenclamide plus EENL group, which was lower (*p* < 0.001) than for the vehicle plus EENL group (Figure 6F) as indicated.

## 4 Discussion

Our results have demonstrated that EENL possesses antinociceptive activity and we explored for the first time some mechanisms underlying this action. Moreover, we have provided further evidence of the lack of toxicity of rambutan fruit peel by using zebrafish embryos.

The evaluation of the toxicity of natural products with therapeutic potential is important to allow translational research. The zebrafish, especially in the earliest development phase, is widely used in pre-clinical tests (Batista-Filho et al., 2021). In the case of EENL, we did not observe toxic effects on zebrafish, both demonstrated by the mortality rate similar to the medium and by the lack of alteration of the embryonic development of zebrafish. The low frequency of abnormalities associated with EENL indicates its high safety.

Corroborating our data, another study showed that the LD_50_ of the hydroethanolic extract of rambutan rind in rodents was higher than 5 g/kg after acute administration, and in sub-chronic studies with doses up to 1 g/kg, no mortality was detected (Thinkratok et al., 2014). Wanlapa et al. (Wanlapa et al., 2015) reported that rambutan flour husks are safe for application in food. They observed that the minimum lethal dose in rats was greater than 2 g/kg. Taken together, these findings suggest the relative safety of rambutan peel.

These results of the lack of toxicity are encouraging regarding the biological properties of EENL, especially considering the chemical composition of this extract. Our data from the phytochemical analysis showed that EENL contains procyanidin B (an anthocyanin), (Epi)-catechin and ellagic acid and its derivatives punicalin, pedunculagin, and ellagic acid-4 (Mahmood et al., 2018)-O-xylopyranoside. The detection of these compounds in EENL is in agreement with previous studies (Thitilertdecha and Rakariyatham, 2011; Hernández et al., 2017). However, we did not detect geraniin and corilagin in our extract, while they have been described as usually found in rambutan extracts. Thus, the presence of secondary metabolites in *N. lappaceum* fruit peel depends on a variety of factors, like the geographic location, chemical composition of the soil, environmental conditions, and stages of fruit development (Thitilertdecha and Rakariyatham, 2011; Monrroy et al., 2020).

We found that the compounds detected in EENL are polyphenols and these compounds have been widely described as able to act against inflammation and possibly pain. It is known that inflammatory processes are commonly associated with pain as a result of the production of inflammatory mediators, which stimulate peripheral and central nociceptive pathways (Baral et al., 2019). Thus, we focused on the evaluation of EENL in models of nociception.

First, we observed that the pretreatment with EENL reduced the number of abdominal writhes in mice after intraperitoneal administration of acetic acid. The antinociceptive effect of EENL in this model is in line with the demonstration that pretreatment with either ellagic acid (Rogerio et al., 2006) or epicatechin (Lopes et al., 2010) reduced the number of abdominal writhes induced by acetic acid in mice.

These observations suggest that EENL may act in nociception by reducing the stimulation of terminal nerves through decreased production of inflammatory mediators. To better understand this effect, we administered EENL to mice that were submitted to formalin-induced nociception and observed that EENL reduced the paw licking/biting time in both the first and second phases of the formalin test. This test is widely used in the search for analgesic and anti-inflammatory drugs, and it is characterized by a biphasic response. The first phase is of short duration and arises from direct stimulation of the sensory C fibers. The second phase is characterized by the release of inflammatory mediators that stimulate nociceptors (Hunskaar and Hole, 1987). Corroborating our data, oral administration of ellagic acid also exhibited antinociceptive activity in both the first and second phases of the formalin test (Nascimento et al., 2011). In addition, Lopes et al. [32] showed that epicatechin decreases the nociceptive response in the second but not the first phase of the formalin test.

Based on second-phase formalin test data, we decided to further test the effect of EENL in a typical model of inflammatory pain, which is caused by carrageenan, an unspecific agent. We found that the administration of EENL reduced the mechanical allodynia caused by carrageenan in mice paws. In agreement with this fact, ellagic acid produced an anti-inflammatory effect in the model of carrageenan-induced paw edema (Rogerio et al., 2006) which corroborates with the reduction of inflammatory pain by EENL administration.

Complementarily, to investigate whether EENL could act on pain caused by direct activation of TRPV1 receptors on sensory fibers, we used capsaicin-induced paw nociception. Capsaicin activates C and Aδ fibers in afferent neurons by stimulating TRPV1 receptors, which leads to the influx of Ca^2+^ and Na^+^, triggering pain (Abdel-Salam and Mózsik, 2023). We demonstrated that EENL diminished the paw licking/biting time in this model, which is in line with the study by Lopes et al. (Lopes et al., 2010) who reported that epicatechin reduced the licking/biting time in the capsaicin test.

Based on our data from abdominal writhing, the second-phase formalin test, and carrageenan-induced mechanical allodynia, we suggest that EENL may reduce the stimulation of sensory fibers by decreasing endogenous algogenic factors. However, this extract may act on other mechanisms of pain, as indicated by the results of the first-phase formalin test. Supporting this assumption, the administration of EENL reduced the heat-induced nociceptive behavior of mice on the hot plate. This test is considered sensitive to drugs that act by modulating the spinal and supraspinal pain response through thermal stimulation of nociceptors, mainly in non-myelinated C fibers (Jacob and Ramabadran, 1978). In this way, our data suggests that EENL promotes antinociceptive effects by acting in the spinal and supraspinal sites of the nociceptive pathways.

In the next experiments, we conducted a mechanistic investigation of the antinociceptive effect of EENL by using pharmacological tools. A growing body of evidence points to NO as a relevant mediator of nociception, although its participation is subject to much controversy (Miclescu and Gordh, 2009; Schmidtko, 2015). The effect of NO synthase inhibitors can be both pro and antinociceptive, depending on the location of the NO formation. In the spinal cord, NO contributes to the processing of nociceptive signals and NO synthase inhibitors usually cause an antinociceptive effect (Schmidtko, 2015), but in the periphery, mainly in models of inflammatory nociception, NO synthase inhibitors may cause different outcomes (Gomes et al., 2020).

In the hot plate test, we found that pretreatment with L-arginine, a NO precursor, did not change the reaction time, but the pretreatment with L-NAME, an unspecific NO synthase inhibitor, increased this time and its effect was blocked by L-arginine, which is partly in agreement with the contribution of NO to nociception and has already been described in other studies (Saad et al., 2016). The antinociceptive effect of EENL was also reversed by L-arginine, indicating the participation of NO, as has been described by others (Sheikholeslami et al., 2021). In particular, (Mansouri et al., 2014), suggested that NO is involved in the antinociceptive activity of ellagic acid in a model of abdominal writhing. Some previous studies have reported that ellagic acid has a potent inhibitory effect on NO production (Umesalma and Sudhandiran).

Additionally, we found that the antinociceptive effect of EENL takes place with the participation of K_ATP_ channels since it was reversed by treatment with glibenclamide, a blocker of these channels (Perimal et al., 2011). The opening of the K_ATP_ channels is important for antinociception because it leads to the hyperpolarization of cell membranes, resulting in decreased cellular excitability (Afify et al., 2013). Likewise, the participation of the opioid system was indicated by the fact that naloxone reversed the antinociceptive effect of EENL and morphine. Morphine is known as an agonist of opioid receptors (Calixto et al., 2000), which are widely distributed in the central nervous system and peripheral tissues of neural and non-neural origin and can be blocked by the administration of naloxone, a non-selective opioid receptor antagonist (Perimal et al., 2011). Interestingly, a previous study showed the involvement of the K_ATP_ channel and opioid receptors in the antinociceptive activity of ellagic acid, a constituent detected in EENL, but in a model of abdominal writhing (Mansouri et al., 2013). Considering that the blockage of opioid receptors, NO synthases, and K_ATP_ reversed the antinociceptive effect of EENL it is reasonable to speculate that these factors are involved in a sequential pathway, rather than parallel mechanisms. To support this assumption, it is worthwhile mentioning that accumulating evidence correlates the antinociceptive effects of opioids with the NO/cGMP/K_ATP_ channels pathway (Ferreira et al., 1991; Dal et al., 2006; Miclescu and Gordh, 2009; Gomes et al., 2020)

An important bias while investigating the antinociceptive effects of natural products is the possibility of central nervous system depression or muscle relaxation, which can alter the motor coordination response and invalidate the results of nociceptive tests (Ol et al., 2020), such as abdominal writhing, formalin, and hot plate reaction. However, we showed that the administration of the highest dose of EENL used in our study did not change the distance covered in the open field, supporting the hypothesis that the observed antinociceptive effect of EENL does not result from interference in the mice’s ability to perform the nociceptive behavior.

## 5 Conclusion

Our results demonstrate that EENL possesses antinociceptive effects, which take place with the participation of opioid receptors, NO, and K_ATP_ channels, and might have a correlation with its constituents, such as ellagic acid. Furthermore, EENL does not alter the mortality or embryonic development of zebrafish.

## Data Availability

The raw data supporting the conclusion of this article will be made available by the authors, without undue reservation.
