# Supplementary material for: Antinociceptive effect of Nephelium lappaceum L. fruit peel and the participation of nitric oxide, opioid receptors, and ATP-sensitive potassium channels
Source: Front Pharmacol. 2023 Oct 31;14:1287580. doi: 10.3389/fphar.2023.1287580 (PMC10644719; doi:10.3389/fphar.2023.1287580)
Supplement: Supplementary file 1 [file DataSheet1.PDF]

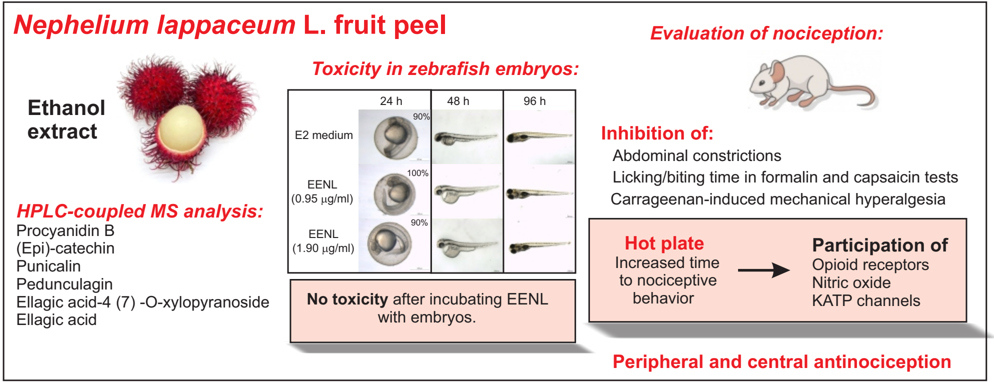

Nephelium lappaceum L. fruit peel
Ethanol extract
HPLC-coupled MS analysis:
Procyanidin B
(Epi)-catechin
Punicalin
Pedunculagin
Ellagic acid-4 (7) -O-xylopyranoside
Ellagic acid
Toxicity in zebrafish embryos:
24 h
48 h
96 h
E2 medium
90%
EENL (0.95 µg/ml)
100%
EENL (1.90 µg/ml)
90%
No toxicity after incubating EENL with embryos.
Evaluation of nociception:
Inhibition of:
Abdominal constrictions
Licking/biting time in formalin and capsaicin tests
Carrageenan-induced mechanical hyperalgesia
Hot plate
Increased time to nociceptive behavior
Participation of
Opioid receptors
Nitric oxide
KATP channels
Peripheral and central antinociception
